# Mood food: antidepressant effects of culinary spices

**DOI:** 10.3389/fnut.2026.1790721

**Published:** 2026-02-25

**Authors:** Lu Zhong, Yingjie Qing, Jie Liu

**Affiliations:** 1 State Key Laboratory of Technologies for Chinese Medicine Pharmaceutical Process Control and Intelligent Manufacture, Nanjing University of Chinese Medicine, Nanjing, China; 2 School of Pharmacy, Nanjing University of Chinese Medicine, Nanjing, China; 3 Department of Pharmacy, Nanjing Pukou People’s Hospital, Liangjiang Hospital, Southeast University, Nanjing, China

**Keywords:** antidepressant effects, culinary spices, depression, dietary interventions, functional foods

## Abstract

Depression represents a major contributor to the global disease burden. As a complementary strategy, dietary interventions have attracted increasing interest in mental health care. Culinary spices, traditionally used as flavoring agents, are now increasingly regarded as functional foods due to they contain diverse plant-derived bioactive compounds with neuroprotective potential. Growing evidence suggests that several commonly used spices, such as turmeric, chili pepper, black pepper, ginger and saffron, all show similar antidepressant effects. These effects are achieved by regulating neuroinflammation, oxidative stress, monoaminergic neurotransmitter, neuroplasticity and gut-brain axis. Different from traditional antidepressants, culinary spices are typically consumed as a part of daily diet in low doses over extended periods, which may allow for gradual biological effects through multiple pathways while maintaining a favorable safety profile. This review systematically summarizes the evidence of antidepressant effects of major culinary spices, explains the molecular mechanisms, and discusses the key issues related to bioavailability, safety and dietary therapy potential. Understanding the role of culinary spices in emotional regulation may provide valuable insights for nutrition-based depression prevention and auxiliary management strategies.

## 1 Introduction

Depressive has become the main causes of global disease burden, causing great disability and health problems worldwide (1). The most common approach is to first administer psychotherapy, followed by antidepressant medication (2, 3). For antidepressant medication of outpatients with depression, doctors usually take selective serotonin reuptake inhibitors (SSRIs) as the first choice (4, 5). Although antidepressants therapy, is still the main method for major depressive disorder (MDD), rates of remission are still unsatisfactory (6). At present, antidepressant treatment methods are often limited, such as slow onset, ineffective use by a considerable number of people (part of patients are treatment-resistant), and various adverse side effects, from weight gain to sexual dysfunction (7–10). These limitations, coupled with the recurrent nature of emotional disorder itself, urge everyone to urgently find a safe, accessible and effective auxiliary strategy to strengthen the standard treatment.

In this context, nutritional psychiatry, as a new field, has attracted more and more attention (11). It studies the complex relationship between our eating habits and mental wellbeing (12). In this framework, culinary spices, such as turmeric, saffron and ginger, used to be thought of as condiments, but now more and more people realize that they are actually concentrated sources of bio-active phytochemicals, which have a good protective effect on the brain (13–15). These spices are rich in polyphenols, alkaloids, and terpenes, which have been used for hundreds of years in traditional Chinese medicine to help relieve melancholy and restore emotional balance (16–19). Unlike those synthetic drugs that usually target only one receptor, these “Mood Foods” seem to be able to play multiple roles at the same time, such as regulating body’s inflammation, oxidative stress, and gut microbiome (20–22).

In this review, we want to systematically sort out the evidence about the antidepressant effects of five main culinary spices: turmeric, saffron, ginger, chili pepper and black pepper. We will study how they can help the brain to restore balance, such as regulating monoamine nerve transmission, making the hypothalamic-pituitary-adrenal (HPA) axis work normally, and improving neuroplasticity through brain-derived neuropathic factor (BDNF). In addition, we will also discuss a special pharmacological concept called “food synergy”-specifically, some spice combinations (such as turmeric and black pepper used together) can be better absorbed by the body-and what practical difficulties will be encountered in applying these spice therapies to modern psychiatric treatment.

## 2 Modulating the depressed brain: key pathways

**FIGURE 1:**
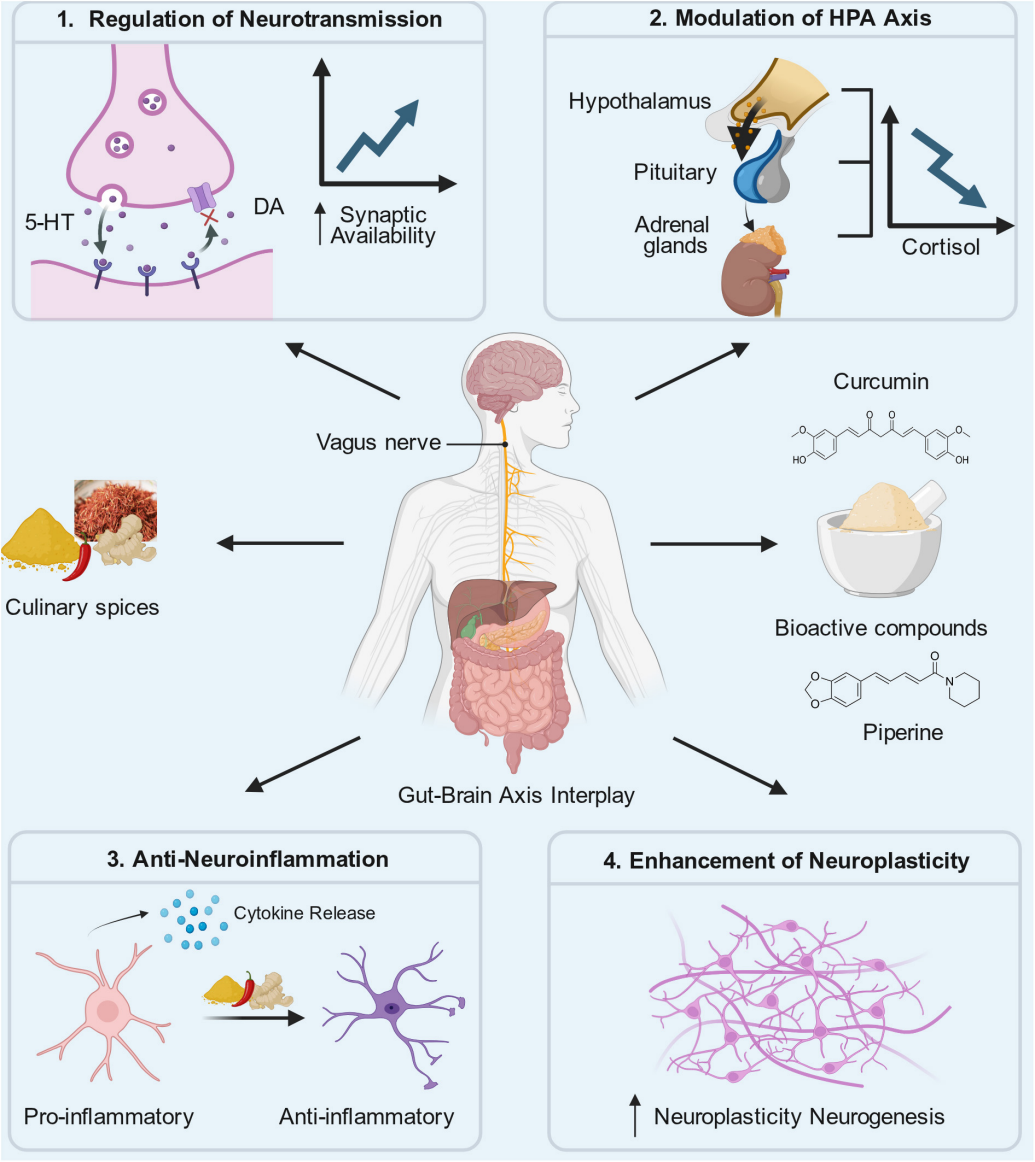
Multilevel mechanisms underlying the antidepressant effects of major culinary spices.

Before discussing individual culinary spices, we first outline the major neurobiological pathways involved in depression that are potentially modulated by dietary bioactive compounds (Figure 1).

### 2.1 Regulation of monoaminergic neurotransmission

According to the monoamine hypothesis, depression is due to the lack of key neurotransmitters in the brain, mainly 5-hydroxytryptamine (5-HT), dopamine (DA) and norepinephrine (NE) (23). When our bodies work normally, 5-HT is responsible for managing our emotions, sleep and appetite (24). NE controls whether we are awake, energetic and how to deal with stress (25); and DA is especially important for the rewards system, the motivation to do things and being happy (26). Therefore, if these signal pathways are confused, the MDD will appear, such as being depressed all the time, slow in action and thinking, and not being interested in anything. The amount of these monoamine substances in synapses is strictly controlled by recycling transporters and degrading enzymes such as monoamine oxidase (MAO) (27). Therefore, treatment methods-including traditional antidepressants and active natural compounds-usually try to restore the balance of monoamine system by inhibiting their recycling or preventing them from being degraded by enzymes.

### 2.2 Modulation of the hypothalamic-pituitary-adrenal axis

The hyperactivity of HPA axis and the subsequent increase of serum cortisol are typical features of MDD (28–30). The imbalance of HPA axis is mainly manifested by long-term high cortisol, which plays a key role in the pathogenesis of depression (30). Although cortisol is very important for acute stress response, long-term exposure to high levels of cortisol will produce harmful neurotoxicity to the central nervous system (31). Hippocampus is the main target of this toxicity (31). The increase of cortisol caused by long-term stress will reduce the level of BDNF and inhibit the nerve regeneration of adult hippocampus (32). This will lead to structural changes, including the shrinkage of hippocampus and the decrease of synapses, which are clinically related to the cognitive impairment and emotional instability of MDD patients (33).

### 2.3 Anti-neuroinflammation and regulation of glial network

Now there is more and more evidence that depression is actually a systemic inflammatory problem, people with MDD have a higher comorbidity rate with immune and inflammatory diseases such as rheumatoid arthritis, metabolic syndrome, and inflammatory bowel disease (34–36). Psychosocial stress triggers alterations in hematopoiesis—specifically inducing monocytosis, neutrophilia, and lymphocytopenia—which subsequently upregulate pro-inflammatory processes in peripheral tissues via autonomic and neuroendocrine pathways. This uncontrolled peripheral inflammation will not always be confined to the same place. Pro-inflammatory cytokines produced by innate immune system can cross the blood-brain barrier (BBB) and transform immune signals of the whole body into central nervous system inflammation. This process may only be manifested as “sick behavior” at first, but in those who are easily affected and have been in an inflammatory state for a long time, it will cause depressive symptoms (37, 38). In other words, inflammation in the body will cross the BBB and affect the work of our neurons.

The core problem of this neuropathy is that microglia, the main immune cells in our brain, are not working well. Now there is even a view that depression can basically be said to be a “microglia disease” (39). Microglia are super important, they are responsible for regulating inflammation, helping nerve connection changes and brain network formation. However, under long-term pressure, microglia will be pathologically “activated,” and this process is mainly controlled by P2 × 7 receptor (P2 × 7R) (40). Once activated, these cells will release many proinflammatory cytokines, especially Interleukin-6 (IL-6). Crucially, the dysfunction of this microglia will lead to a series of neurotoxicity: the IL-6 secreted by it will directly lead to the death and atrophy of astrocytes-and the problem of astrocytes is another key sign of major depression. Therefore, the release of IL-6 mediated by P2 × 7R is a specific path through which activated microglia destroys the glia network, thus causing depression (40, 41).

### 2.4 Enhancement of neuroplasticity and neurogenesis

Neuroplasticity refers to the ability of our brain to change its neurobiological structure according to external stimuli (such as long-term stress) or internal stimuli (mainly the influence of genes or epigenetics) (42). Mature neural stem cells become new neurons in our adult hippocampus. This process is called adult hippocampal neurogenesis, which is a major principle of neuroplasticity (43). The operation of this principle is managed by epigenetic adjustments, which will affect transcription factors, non-coding RNA and metabolic pathways (43).

Depressed patients exhibit disordered neural circuits responsible for mood regulation and cognition (44). Decreased neuroplasticity and atrophy of hippocampal neurons are typical structural features of depression, which usually lead to cognitive impairment in patients (45). BDNF, which is a kind of protein that is essential for neuron survival and synaptic growth, plays a role in promoting brain plasticity by binding with high affinity TrkB receptors on postsynaptic neurons. This combination will start some important downstream signaling pathways, such as PI3K/Akt and mTOR pathways, which will eventually activate a transcription factor cAMP-response element binding protein (CREB). As a commander-in-chief, phosphorylated CREB will promote the expression of genes that are essential for neurite growth, neurogenesis and synaptic plasticity, thus actually strengthening the neural connection (46, 47). Antidepressants can cure diseases mainly by activating cAMP-CREB-BDNF signaling pathway, allowing nerve cells in hippocampus to grow again and repairing damaged neural plasticity (48).

### 2.5 The gut-brain axis interaction

Gut-brain axis is a complex two-way communication network, which connects enteric nervous system and central nervous system (49, 50). It provides a new way for drugs to affect emotions without directly entering the brain. The gut microbiota (GM) is a diverse ecosystem composed of various microorganisms, including bacteria, fungi, and viruses. Under normal conditions, these microbes maintain a symbiotic relationship with the host and are essential for health. Their primary functions include assisting in nutrient digestion, strengthening the intestinal barrier, synthesizing vitamins, and regulating both metabolic and immune responses (51). Depression is often accompanied by GM imbalance (51–53). GM has a great influence on the central nervous system. On one hand, the central nervous system plays a key role in modulating gastrointestinal functions and the composition of the GM, including intestinal motility, secretion, and digestive processes. On the other hand, the gut microbiota can influence brain activity via neural, endocrine, and immune pathways. This bidirectional communication system is known as the microbiota–gut–brain (MGB) axis and represents a critical mechanism through which intestinal microorganisms shape cognitive functions and behavioral outcomes (54). One of the important mechanisms to overcome depression is to change the gut microbiota.

Diet is the key factor to determine the composition and function of GM, which is particularly important in the chain of “diet-microbiota-depression” (55). Mediterranean Diet (MD) has a strong anti-inflammatory effect because it contains a lot of polyphenols and dietary fiber (56). A randomized controlled trial clinical study proved that MD can obviously improve the depressive symptoms of young men and improve their quality of life (57). In principle, this diet can increase the diversity of microbial, reduce intestinal inflammation and make the intestinal barrier stronger. Therefore, it is now recommended to use dietary regimens rich in plant-derived compounds, polyunsharated fatty acids (PUFAs), vitamins and minerals to deal with depression (55). However, it is best to customize a personalized nutrition program to achieve the best therapeutic outcomes.

## 3 Antidepressant-like effects of major culinary spices

### 3.1 Turmeric

Turmeric is one of the most widely consumed spices worldwide, and it is also a well-studied source of dietary polyphenols, in which curcumin is the main active ingredient (58). In cooking, turmeric is the most important part of Indian curry. Turmeric is seldom consumed alone; instead, it is fried with other spices in oils. A large number of animal experiments have found that curcumin has similar antidepressant effects in several commonly used animal models of depression, such as olfactory bulbectomy, corticosterone-induced stress, and monoamine depletion caused by reserpine (59). In the forced swimming test and sucrose preference test, depressive-like behaviors can be steadily improved by administration of curcumin (59, 60).

Mechanistically, curcumin is considered to reduce neuroinflammation and immune-modulating properties, mainly by inhibiting the pro-inflammatory cytokines such as cyclooxygenase-2 and prostaglandin E2 (61). At the same time, it regulates our neurotransmitters and hormones by increasing the levels of serotonin, dopamine and noradrenaline, while reducing glutamate and cortisol (62). Studies have found that curcumin has neuroprotective and neurogenesis effects, mainly by modulating the activity of *bacteroides*, increasing BDNF and 5-HT receptors to reduce the damage (63, 64). Fourthly, it can resist oxidative stress and nitrosative stress, because it can enhance antioxidant defense and reduce nitric oxide (65, 66). Finally, it can protect mitochondria and improve the common metabolic problems of depression (67).

From a nutritional point of view, turmeric is usually eaten as part of the daily diet for a long time. Although the bioavailability of curcumin itself is not high, if it is eaten with black pepper containing piperine, it can improve its absorption by the body, which shows that food collocation is very important for its emotional regulation (68–70).

### 3.2 Saffron

Saffron comes from the dried stamen of *Crocus sativus L* (71). Iran is the largest saffron producer in the world, and 90% saffron in the world comes from there (72). Saffron is the most important ingredient in Spanish paella when it comes to cooking. The water-soluble crocin dissolves into the cooking broth while the dish is being made, coating the rice grains and providing the dish’s characteristic color and smell. This culinary matrix serves as a practical “Mood Food” delivery system that provides bioactive compounds in a stable and accessible for long-term emotional regulation. It has become a promising alternative medicine for the treatment of depression (72, 73). Crocin, crocetin, and safranal are the main bioactive components in it, and scientists have explored the potential therapeutic applications in laboratory and clinical research including antidepressant (74).

Studies have found that saffron and its ingredients can exhibit antidepressant-like behaviors in animal models with chronic restraint stress-induced depression (75, 76). Scientists found that their gut microbiota became richer and more diverse after being treated with crocetin, a component of saffron (77). Importantly, saffron is one of the few spices that have been tested by randomized controlled trials (78–80). Saffron has been shown to have a regulatory effect in improving patients’ quality of life (81). These tests found that eating saffron supplements can significantly improve the symptoms of people with mild to moderate depression.

Studies show that its mechanisms may include regulating neurotransmitters such as serotonin, DA, and NE. The mechanism involves inhibiting serotonin reuptake at the synapse, thereby prolonging the elevation of serotonin levels and enhancing its role in mood regulation (82). Saffron also can regulate the HPA axis by lowering corticosterone, producing neuroprotective efficacy in the brain, and positive effect on BDNF (83). Recently, there is new evidence that it may be related to the inhibition of inflammation and oxidative stress (84). Saffron may affect mood by regulating our gastrointestinal function and systemic inflammation, which shows that its function is related to the “gut-brain axis.” The gut–brain axis is also involved in the pathophysiology of depression, as alterations in GM can influence neuroimmune and neuroendocrine signaling pathways that interact with the brain (77). As a traditional ingredient, saffron is usually used in dietary cuisine and is acceptable to everyone.

### 3.3 Ginger

Ginger is a condiment that we often eat, and there are many bioactive compounds such as gingerols and shogaols in it (85, 86). More and more evidences show that ginger and its active ingredients may have antidepressant effects. In a mouse depression model called chronic unpredictable mild stress (CUMS), 6-gingerol, the main component of ginger, can obviously reduce the depressive behaviors, and it can also inhibit neuroinflammation caused by microglia (87). It is found that 6-gingerol regulates the polarization of microglia by activating peroxisome proliferator-activated receptor γ signal pathway, which may be the key mechanism of its antidepressant (87).

At the same time, another component of ginger, 6-shogaol, can not only improve dyskinesia, but also alleviate depressive behaviors in mouse model of Parkinson’s disease. These effects are closely related to the recovery of monoamine neurotransmitters (such as DA, serotonin and NE) in striatum and hippocampus (88). Generally speaking, these findings prove that ginger can play an antidepressant role by regulating neuroinflammatory response and neurotransmitter balance.

### 3.4 Chili pepper

Chili peppers is a spice that many people all over the world are eating. It contains the primary active alkaloid, capsaicin (89). In addition to its function as pain detectors because of the well-known target of transient receptor potential cation channel, subfamily V, member 1 (TRPV1), which is related to our stress response, inflammation in the brain, and nerve signals that affect emotions, these medications may play a key role in controlling anxiety and other emotional responses (90–93). Some studies found that capsaicin an anxiolytic effect in specific rat models and can protect hippocampal synaptic plasticity and spatial memory recovery through the TRPV1 channel (94, 95). Capsaicin plays an antidepressant role by regulating the connection between intestine and brain. It obviously improved the depressive behaviors, increased 5-HT in blood and decreased tumor necrosis factor α(91). In principle, these benefits were mechanistically linked to the remodeling of gut microbiota composition.

From the point of view of eating, consistent, moderate eating chili pepper can be beneficial for depression and mood. However, excessive intake may irritate the stomach and intestines, so how and how much you eat is really crucial.

### 3.5 Black pepper

Black pepper is one of the most common spices in the kitchen, and it contains a main active ingredient piperine (96). It plays an antidepressant role in two ways, one is HPA axis, and the other is neuroplasticity. In the CUMS rat model, piperine significantly improved the behavior disorder by reducing the levels of adrenocorticotropic hormone and corticotropin-releasing factor in serum, but had little effect on Corticosterone (CORT) (97, 98). In addition, in the cell experiment induced by CORT, piperine restored neuroplasticity by regulating the proliferation, migration and differentiation of neural progenitor cells, which was reflected in the reduction of immobility time in behavior test (98, 99).

Studies have found that piperine has a powerful ability to improve the absorption effect of other plant components (such as curcumin). Indian curry powder (Garam Masala) provides a classic example of food synergy in a real-world dietary matrix. The co-presence of black pepper and turmeric in these traditional spice blends is not merely for flavor. The piperine from black pepper serves as a natural bio-enhancer. Curcumin-piperine co-supplementation can promote bioavailability, reduce oxidative stress and depression (70, 100, 101). Therefore, black pepper may not only have a “refreshing” effect on itself, but also make us feel better by matching with other foods.

## 4 Synergistic effects and bioavailability

Although preclinical studies have found that some ingredients extracted from spices exert neuroprotective effects, a major limitation is their poor bioavailability following administration (102, 103). However, our traditional cooking methods are ingenious, and we often mix different spices together or cook in a specific way, which unintentionally solves these problems. This part is about why eating whole spices directly may be better than eating only the extracted single ingredient, which is behind the help of “food synergy” and “entourage effect.”

### 4.1 The bioavailability challenge

Although dietary polyphenols have strong biological activity, they are characterized by poor oral absorption, which scientists call “low bioavailability/high bioactivity paradox” (104, 105). After eating, these components will undergo complex Phase I and Phase II enzyme metabolism in intestinal cells and liver, and finally less than 5% of the original substances can enter the blood circulation (104, 105). The therapeutic effects of polyphenolic (especially curcumin) are severely limited by their low oral bioavailability (106). Because curcumin is hydrophobic, it is difficult to dissolve in gastrointestinal fluids. Moreover, it is rarely absorbed in the small intestine, then it is rapidly metabolized in the intestine and liver, and then it is quickly excreted through feces, and only a small part is excreted from urine (106, 107). Therefore, even if we take a large dose of pure curcumin orally, the concentration in the blood can hardly be measured, and it can’t reach the therapeutic level needed to protect the brain nerves.

### 4.2 Food synergy: the wisdom of culinary combinations

**FIGURE 2:**
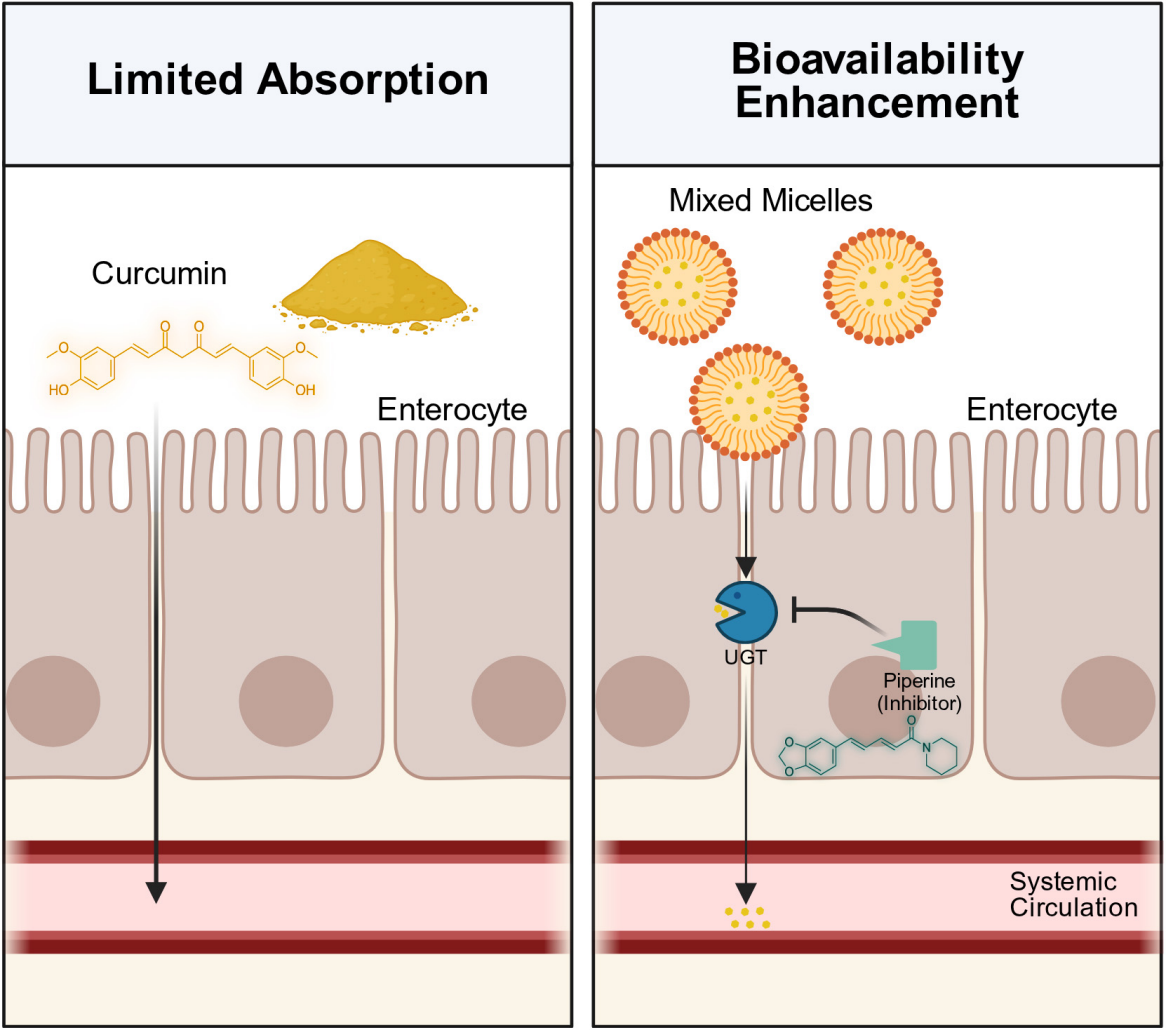
Synergistic effects of dietary lipids and piperine on the intestinal absorption and metabolic stability of curcumin. UGT, uridine diphosphate glucuronosyltransferase.

In everyday diets, multiple spices are often consumed together with dietary fats, thus forming a “food matrix,” which can influence the absorption and bioavailability of bioactive compounds in the body (108, 109). The most reliable example is that turmeric and black pepper are eaten together (110). Piperine, the spicy ingredient in pepper, is a super powerful “absorption helper.” The pharmacokinetic profile of curcumin can be improved by combining it with piperine. Piperine acts by inhibiting the activity of metabolizing enzymes, particularly glucuronidase, in the liver and intestine (111). As a result, the rapid glucuronidation of curcumin is inhibited, thereby reducing its urinary excretion (70) (Figure 2). In addition, piperine modulates membrane dynamics and increases the permeability of the intestinal absorption site (112). A study reported that co-administration with piperine increased the bioavailability of curcumin in the bloodstream by up to 2000% without producing detectable adverse effects (112, 113). When curcumin is used together with piperine, its therapeutic effect is much stronger than that when it is used alone. This synergy makes the levels of serotonin and DA rise higher and the activity of MAO can be inhibited more effectively (114).

Traditionally, spices are often fried in oil. This lipid-rich environment facilitates the components from spices easier to dissolve. Scientifically, this “oily environment” created by traditional cooking conforms to the basic principles of lipid-based drug delivery systems, especially like a Type I lipid formula (115). The design idea is similar to that of modern self-nanoemusifying drug delivery systems, and the main goal is to solve the problem of dissolution (116). By dissolving hydrophobic curcumin in food oil during heating, the food substrate is like a simple transport cart (117). This pre-dissolution ensures that the bioactive compounds we eat enter the gastrointestinal tract in a dissolved state, so that they are more easily emulsified by bile salts in the body and added to the mixed micelles. This traditional culinary practice actually utilizes a mechanism analogous to that of the modern pharmaceutical lipid-based delivery systems to promote bioavailability (116) (Figure 2).

### 4.3 The “entourage effect”: whole spices vs. isolates

Drawing lessons from the paradigm originally established in cannabis research, “entourage effect” proposes that the therapeutic effect of the whole plant matrix exceeds the active ingredients separated from it (118, 119). Although drug development usually gives priority to the extraction and purification of a single “silver bullet” molecule-such as curcumin extracted from turmeric or piperine– extracted from pepper-new evidence shows that the complex phytochemicals in the whole culinary spices provide unique pharmacological advantages. For example, although curcumin is undoubtedly the main neuroprotective agent in turmeric, this rhizome also contains essential oil, and the most important one is aromatic turmerone (ar-turmerone) (120). Recent studies have pointed out that ar-turmerone itself shows biological activity, especially it can the moderation of neuroinflammation and stimulate the proliferation of neural stem cells, which creates an entourage effect (121).

Similarly, saffron is like a complex plant pharmacy, which contains more than 160 volatile and aromatic compounds (72, 122). Its antidepressant effect is not based on a single component, but on the mutual cooperation of multiple bioactive components such as crocin and safranal (72). In addition, the natural plant fiber and other polyphenols in spices are like a protective shell, which can help fragile active ingredients not to be destroyed quickly in the strong acid environment of the stomach-this stability is often lost in purified extracts. Therefore, eating whole spices is a “multi-target” strategy, which may be wider and more stable than single-component supplements.

## 5 Discussion

We should no longer regard the spices in the kitchen as condiments, but they are actually powerful “Mood Foods,” which have many protective effects on the brain. Unlike ordinary antidepressants (such as SSRIs), which generally rely on a single dominant mechanism, bioactive compounds in spices-such as curcumin, crocin, and piperine may exert multiple complementary effects. Together, they can regulate the neuronal communication, reduce neuroinflammation through the gut-brain axis, and enhance brain plasticity. This comprehensive approach is particularly useful for treating depression, because we now know that depression is actually a systemic problem, which is related to metabolism, immunity and endocrine system (123). Spices combinations can help these interconnected systems to restore balance, so they may have advantages over single drugs, especially in improving physical discomfort that often occurs with emotional problems.

To translate these findings into clinical practice, an important consideration is the potential gap between typical culinary intake and the doses that may be needed to elicit therapeutic effects. Some research data show that long-term consumption of spices may be associated with a lower risk of depressive symptoms, suggesting that their inclusion as part of the daily diet could contribute to the maintenance of emotional wellbeing (124). However, those clinical trials that prove that spices can make people feel better quickly often use high-concentration extracts, which can’t be achieved simply by eating at ordinary times (72, 78, 125). Although adding spices to our daily meals can be used as a good preventive method or an auxiliary lifestyle, if a person has moderate to severe depression, adding spices to meals may not be enough as the main treatment. Therefore, future clinical guidelines must distinguish clearly: which are “dietary suggestions” to keep us happy, and which are “nutritional drug prescriptions” to treat diseases.

Although kitchen spices are usually safer than synthetic psychotropic drugs, they are not completely risk-free. This is most evident in the characteristics of capsaicin and other spicy substances-at low doses, they can trigger beneficial stress response and protect neuro, but excessive intake may lead to gastrointestinal mucosal damage and desensitization of sensory neurons, and may even aggravate systemic inflammation (126–128). Moreover, the long-term antidepressant effect of some spices may be affected by physical tolerance. For example, long-term high-frequency exposure to capsaicin can trigger TRPV1 receptor desensitization, which lead to the weakening of the signal pathway of emotional regulation and neuroprotection in response to stimuli (129). Theoretically, this desensitization phenomenon may gradually weaken the effect of pepper on stabilizing mood. Therefore, we need more research to determine the best dietary intake, which can not only maintain receptor sensitivity, but also not affect the long-term therapeutic effect.

In addition, some specific interactions need clinical attention, for example, high dose saffron may lead to blood toxicity due to its antiplatelet activity (130). Curcumin and piperine, the core components of the “Mood Food” synergy, are known to modulate key metabolic enzymes. Specifically, piperine is a potent inhibitor of cytochrome P450 (CYP) enzymes (131). Since many standard antidepressants, including SSRIs and tricyclic antidepressants, are extensively metabolized in the liver by CYP450, co-administration with spice extracts may theoretically elevate the plasma concentration of these drugs, increasing the risk of dose-dependent toxicity (132, 133). Furthermore, curcumin’s inhibition of certain CYP isoforms and UDP-glucuronosyltransferase enzymes could further complicate the metabolic clearance of psychiatric medications (134, 135). Moreover, to enhance the clinical utility of spice-based interventions, specific contraindications must be identified. For example, people who have bleeding problems or are taking anticoagulant drugs should be especially careful when avoiding or using saffron due to its antiplatelet activity (130). High-dose capsaicin should be avoided by patients with active peptic ulcers or severe gastroesophageal reflux disease. Therefore, doctors must carefully evaluate the individual factors of patients, including existing complications and drugs being taken to avoid adverse herbal-drug interactions. A major limitation of diet therapy is that the variability of bioactive content. It depends on its place of origin, harvest time, and storage conditions. Unlike those standardized drugs, this fluctuation makes it difficult for us to ensure a stable antidepressant effect through diet alone. In order to solve this problem, future strategies should focus on the use of standardized spice powders, so that the therapeutic effect can be more stable. In addition, because many polyphenols are poorly absorbed by the body after oral administration, this field must turn to the study of advanced delivery systems. The application of nanotechnology (such as nano-curcumin) and phospholipid complex technology (phytosomes) has shown good prospects, which can bypass liver metabolism and enhance the ability to cross the BBB (136–138). If “Mood Foods” is to be successfully applied in clinic, it is ultimately necessary to combine traditional dietary wisdom with modern pharmaceutical technology, so as to provide accurate, easily absorbed and standardized dosage.

In summary, the spices used in our kitchen are like a natural “happy treasure house.” They are easy to get and inexpensive, and everyone is used to using them. Spices such as saffron, turmeric and pepper link ancient dietary wisdom with modern brain science and provide a great new way for our mental health.

## 6 Conclusion

In a word, culinary spices are a promising, easy-to-obtain and multi-target method to help anti-depression. Different from those with single target, these “Mood Foods” can simultaneously regulate neuroinflammation, HPA axis and MGB axis, resulting in a synergistic effect. Looking forward to the future, integrating this ancient dietary wisdom into the overall “lifestyle medicine” method can provide us with a safer and more cost-effective path to reduce the burden of mental health disorders around the world.
